# The Effect of Ultrasonic Scaling and Air-Powder Polishing on the Roughness of the Enamel, Three Different Nanocomposites, and Composite/Enamel and Composite/Cementum Interfaces

**DOI:** 10.3390/nano11113072

**Published:** 2021-11-15

**Authors:** Ksenia Babina, Maria Polyakova, Inna Sokhova, Vladlena Doroshina, Marianna Arakelyan, Alexandr Zaytsev, Nina Novozhilova

**Affiliations:** 1 Department of Therapeutic Dentistry, I.M. Sechenov First Moscow State Medical University (Sechenov University), 119991 Moscow, Russia; polyakova_m_a@staff.sechenov.ru (M.P.); sokhova_i_a@staff.sechenov.ru (I.S.); doroshina_v_yu@staff.sechenov.ru (V.D.); arakelyan_m_g@staff.sechenov.ru (M.A.); novozhilova_n_e@staff.sechenov.ru (N.N.); 2 Institute of Linguistics and Intercultural Communication, I.M. Sechenov First Moscow State Medical University (Sechenov University), 119991 Moscow, Russia; zaytsev_a_b@staff.sechenov.ru

**Keywords:** surface roughness, ultrasonic scaling, air-powder polishing, nanocomposite resins, composite/enamel interface, composite/cementum interface

## Abstract

We aimed to assess the effects of ultrasonic scaling and air-powder polishing on the roughness of enamel, three nanocomposites (Premise, Herculite Ultra, Harmonize), and composite/enamel and composite/cementum interfaces. Class V cavities were restored in 99 extracted third molars with one of the three nanocomposites and treated with ultrasonic scaler or air-powder polishing device (calcium carbonate or sodium bicarbonate powders). The roughness (Ra) of the investigated surfaces was measured with contact profilometer before and after treatment. The data were analyzed using repeated measures ANOVA. Specimens’ Ra values before instrumentation were near the clinically acceptable 0.2 μm threshold. All techniques increased the roughness of the tested surfaces; however, the enamel was slightly affected. The mean Ra values after prophylaxis for composite, composite/cementum and composite/enamel surfaces were 0.32–0.55, 1.33–1.73, and 1.25–1.36, respectively. The extent of composite surface damage was material dependent. Premise surface was not altered by ultrasonic scaling significantly. Air-powder polishing with both powders produced a greater increase in surface roughness of composite resin and restorations margins than ultrasonic scaling. The Ra values after both types of air polishing for Herculite Ultra and Harmonize were approximately 1.5 and 2 times higher, respectively, than those after ultrasonic scaling (*p* < 0.05).

## 1. Introduction

Periodontal disease is one of the six most prevalent noncommunicable conditions [1,2], affecting between 20% and 50% of people worldwide [3]. Periodontal diseases are a set of inflammatory conditions that affect the supporting structures of the teeth, result in attachment and bone loss, and, without due treatment, can lead to spontaneous tooth loss or extraction [4]. They are associated with the presence of a microbial biofilm, which is a highly organized community of microorganisms embedded in an extracellular matrix composed of polysaccharides [5,6,7,8].

This necessitates periodontal therapy and/or maintenance that involves the removal of supra- and subgingival biofilm and calculus and obtaining a biologically acceptable root surface [4,9]. Biofilm and other dental deposits should be regularly removed every 3 to 6 months [10,11], depending on the patient’s periodontal status.

Oral debridement typically involves ultrasonic/sonic scaling, air-powder polishing, or their combination followed by the application of polishing pastes [12]. Polishing is believed to be an important step, since it reduces surface roughness and marginal seepage [13] and removes stains and biofilm [4].

Ultrasonic scaling has been shown to be less time-consuming and easier to use than hand instrumentation [14,15], although both methods yield similar results [16,17]. Ultrasonic scaling allows effective disruption of biofilms without traumatizing surrounding structures [18].

Air-powder polishing is mostly aimed at eliminating dental plaque and pigmented stains [19]. It causes less operator fatigue and is more time-saving and efficient than the use of abrasive pastes and rubber cups in terms of dental plaque removal from hard-to-reach areas [11,20,21]. Air-powder polishing involves the use of a slurry of abrasive powder, water, and compressed air, which is blasted onto the tooth surface [22,23]. A wide array of powders are available, with sodium bicarbonate, calcium carbonate, and glycine being the most commonly used ones [22]. They may differ with respect to particle characteristics such as shape [24], size, and hardness [11].

However, both ultrasonic scaling and air-powder polishing have several limitations. They may increase hard tooth tissues wear and surface roughness of restorations [13] and promote gap formation at the composite restoration-tooth interface [20]. This results in plaque accumulation under and around restorations, which, in turn, may aggravate periodontal disease [15]. Moreover, surface roughness adversely affects the esthetic appearance and longevity of restorations [16,25].

Composite materials may differ in their susceptibility to surface damage as they contain different resins and filler particles varying in composition, shape, and size [11]. Particle size affects the roughness of resin composite restorations and surface hardness [13]. Nanocomposites are widely used in restorative dentistry due to their excellent esthetic characteristics, mechanical strength, and relatively low polymerization shrinkage [26,27,28]. They contain particles ranging in size from 5 to 100 nm, which guarantees surface gloss and smoothness over long periods of time [26,29]. Moreover, composites containing nanosized particles leave less interparticle distance, better protecting resin matrix against wear, and are thus more wear resistant [30]. However, various dental prophylaxis techniques can affect composite surface properties and cause degradation and staining [13].

Composite surface roughness is an important, but not the only, factor to be considered. The quality of restoration margins is also essential for treatment outcome, since inadequate marginal integrity increases the risk of recurrent caries and periodontal disease [31]. As dental plaque and calculus are mostly present in the cervical area of teeth, restorations of Class V cavities are inevitably exposed to periodontal maintenance procedures [14,15,32]. The gingival margins of such restorations are close to the periodontal tissues; therefore, the marginal integrity of cervical restorations may be altered during ultrasonic scaling or air-powder polishing [16,18].

While many studies have described the effects of periodontal therapies on oral hard and soft tissues [4,12,24,33,34,35], and several studies have assessed their effects on composite surface roughness and marginal gap formation, to the best of our knowledge, few studies have evaluated both ultrasonic scaling and air-powder polishing in the framework of a single in vitro research.

Therefore, we aimed to assess the effects of ultrasonic scaling and air-powder polishing on the roughness of the enamel, three different nanocomposites, and composite/enamel and composite/cementum interface.

The null hypotheses tested were that there would be no differences in surface roughness (1) among the composite resins treated using different hygienic procedures, (2) among the three hygienic procedures for each composite resin and enamel, or (3) among the investigated surfaces, i.e., the composite surface, composite/enamel interface, and composite/cementum interfaces.

## 2. Materials and Methods

### 2.1. Specimen Preparation

We selected 99 caries-free human third molars for this study. Their use was approved by Sechenov University’s Ethics Committee (No. 0417, 17 April 2017). The teeth were extracted in the context of the treatment plan, and all patients gave written informed consent. After the extraction, the teeth were scaled to remove organic and inorganic debris and stored in 0.1% thymol solution [4,22,36] for 1 week for disinfection. To prevent dehydration, the samples were kept in distilled water until they were used [37,38]. All procedures were performed with the use of a dental operational microscope (OPMI PROergo, Carl Zeiss Meditec AG, Oberkohen, Germany). The presence of any defects was evaluated during the preparation of the specimens, and specimens with scratches and cracks were excluded.

The teeth were mounted individually in plastic cylinders embedded in a self-curing acrylic resin (Rebaron, GC Corporation, Kasugai, Japan), exposing the buccal surface. Class V cavities with a width of 4 mm, length of 2 mm, and depth of 2 mm were prepared on the buccal surfaces with the 801–010 medium diamond bur (Hager & Meisinger GmbH, Neuss, Germany) in a high-speed handpiece under air/water cooling. The 2 mm bevel was prepared at the occlusal margin of the cavity, and the gingival margin was located strictly on the cementoenamel junction.

Samples were randomly divided into 3 groups for further restoration using different A2-shade nanocomposite resins (Premise, Herculite Ultra, and Harmonize; 33 samples per composite). Table 1 shows the resin composites used in our study and their compositions.

Phosphoric acid (37%) was applied for 30 s on the enamel and 15 s on the dentine, then rinsed off with water for 60 s. The enamel and dentine were dried using the air from the air/water syringe. The three-step adhesive system OptiBond FL (Kerr, Scafati, Italy) was used: primer was applied on the dentine for 15 s followed by the application of gentle air stream to evaporate the solvent. OptiBond FL adhesive was applied on the enamel and dentine for 15 s. The excesses were removed with a microbrush, and the adhesive layer was light cured. The polymerization procedure was carried out using the Demi Plus LED light-curing system (Kerr, Middleton, WI, USA). The Demi Plus is a high-power system (1100–1330 mW/cm^2^), and in accordance with the manufacturer’s recommendations, the adhesive layers and enamel composite A2-shades were cured for 5 s.

The restorative materials in each group were manipulated according to the manufacturer’s instructions and placed into the prepared cavity. The cured samples were then stored in distilled water at 37 °C for 24 h prior to final surface treatment [39].

All specimens were preliminarily finished with the 858F–014 fine diamond bur (Hager & Meisinger GmbH, Neuss, Germany) and polished with a series of aluminum oxide abrasive discs (OptiDisc, Kerr, Bioggio, Switzerland) with a contra-angle handpiece (SMARTmatic S20; KaVo, Biberach, Germany) at a speed of 10,000 rpm for 10 s with a medium grit (40 μm) disc, and at a speed of 30,000 rpm for 10 s with fine grit (20 μm) and extra-fine grit (10 μm) discs [19]. The specimens were stored for 24 h in distilled water at 37 °C prior to ultrasonic scaling or air-powder polishing [13,19].

Before any treatment was applied, each composite specimen was subjected to the first rugosimetric reading in 3 different areas: composite surface, composite/enamel interface, and composite/cementum interface. Thirty-three randomly chosen specimens were used for enamel roughness assessment. After these first readings, all specimens received one of the following prophylactic treatments: ultrasonic scaling, air-powder polishing using sodium bicarbonate, and air-polishing using calcium carbonate.

A third of the specimens (*n* = 33) were ultrasonically scaled using a SATELEC (Satelec Acteon Group, Bordeaux, France) piezoelectric scaler with a N1 tip under water cooling, 10 cycles for 10 s each, at a low power setting (level 3). The number of cycles was determined based on the average service life of the restoration (no less than 5 years), since professional oral hygiene care is usually performed biannually. The scaler tip was angled at 15–20° to the restoration surface and was moved from the gingival margin to the coronal aspect of the restoration.

Other specimens were air-polished by PROPHYflex 3 (KaVo, Biberach, Germany), 10 cycles for 10 s each, at 4 bars of pressure at a 60-degree angle and 5.0 mm away from the surfaces. The number of cycles was determined as mentioned above. Two types of powder were used, sodium bicarbonate powder with particle size 60 µm–70 µm (PROPHYflex™ Powder, Kavo, Biberach, Germany, *n* = 33) and calcium carbonate powder with particle size 60–70 μm (PROPHYpearls™ Powder, Kavo, Biberach, Germany, *n* = 33).

After each prophylaxis cycle, all specimens were polished with Cleanic paste (Kerr, Bioggio, Switzerland), RDA = 27. The paste was applied with a prophylaxis brush with nylon bristles (Pro-Brush™, Kerr, Bioggio, Switzerland) rotating at a low speed for 10 s.

To prevent operator variability, all instrumentations were performed by one operator who was not aware of the type of composite material. The specimens were rinsed in running tap water for 30 s, cleaned in an ultrasonic bath for 5 min, air dried for 20 s, and submitted to a new rugosimetric reading as described below.

### 2.2. Surface Roughness Measurement

The surface roughness (Ra) of the specimens was determined before and after ultrasonic scaling and air-powder polishing, and differences were measured using a profilometer.

The specimens in each group were rinsed for 30 s, after which they were dried with air/water syringe, and surface roughness was evaluated in terms of Ra value (μm) using a Surface Roughness Tester (Mitutoyo, Tokyo, Japan, Surftest SJ-410), with stylus moving at a speed of 0.1 mm/s. The start point for the measurements was marked on the samples. For this purpose, two holes were drilled into the surface to serve as reference points. One located on the enamel 3 mm coronally to the restoration and the other located on the cementum 3 mm apically to the restoration. The line joining these points was used as a reference from which the measurements were performed in perpendicular direction within investigated areas. Ten tracings were performed, 0.8 mm in length each, in the following zones: enamel, composite surface, composite/enamel interface, and composite/cementum interface. Then the mean values were calculated.

### 2.3. Statistical Analysis

Sample size was calculated based on the study by Güler et al., with similar design. The means for the two groups of different nanocomposites after air abrasive polishing in the aforementioned study were 0.453 and 0.408, with a standard deviation of 0.08 [39]. The calculation yielded sample size 99 (power = 80%, type I error rate 0.05, with the adjustment for post hoc multiple pairwise comparisons), meaning 11 samples per group. The normality of distribution and equality of variances were assessed using the Shapiro–Wilk test and Levene’s test, respectively. The Ra data were presented as means and standard deviations for each group. A repeated measures mixed ANOVA test was performed followed by a post-hoc Tukey HSD test for independent groups and a repeated measures t-test with adjustment for multiple comparisons for dependent groups.

## 3. Results

Ninety-nine teeth were randomly assigned to the treatment groups (11 samples for each composite treated with one of the following hygienic procedures: ultrasonic scaling (USS), air-powder polishing using sodium bicarbonate (APPSB), or air-powder polishing using calcium carbonate (APPCC)). The pretreatment and post-treatment Ra values for each group are presented in Table 2. Out of these 99 samples, 33 (i.e., 11 per each prophylaxis treatment) were used for the assessment of enamel roughness (Table 3).

### 3.1. Composite Surface

Composite surface roughness did not differ significantly among composites at baseline (*p* > 0.05). There was a significant increase in Ra values after hygienic treatment with USS, APPCC, and APPSB in all composite groups (*p* < 0.05), except for the Ra values in the Premise group after USS (*p* = 0.092). Besides, Ra values after both types of air-powder polishing for Herculite Ultra and Harmonize were approximately 1.5 and 2 times higher, respectively, than those after USS (*p* < 0.05). Harmonize exhibited the roughest surface among all composites after all hygienic procedures.

### 3.2. Enamel

Initial enamel roughness did not differ significantly among the groups and ranged between 0.55 μm and 1.33 μm. Hygienic treatment was a significant factor for enamel roughness (*p* = 0.0003). There was a slight increase in surface roughness in all groups, although only in the APPSB group enamel surface roughness was significantly higher than baseline measurements: the mean values were 1.40 ± 0.31 μm and 1.13 ± 0.29 μm, respectively (*p* = 0.0469). The differences between Ra values after USS and APPCC treatment were insignificant compared with baseline (*p* = 0.0583 and *p* = 0.0834, respectively).

### 3.3. Composite/Enamel Interface

Composite/enamel interface roughness did not differ significantly among the composites at baseline (*p* > 0.05). There was a significant increase in Ra values after hygienic treatment with USS, APPSB, and APPCC in all composite groups (*p* < 0.05), except for the Ra values in the Premise group after USS (*p* = 0.878). Herculite Ultra showed the roughest surface among the composites after USS, although the differences were insignificant. Harmonize/enamel roughness values were significantly greater than that of Premise/enamel after APPSB and APPCC treatment.

### 3.4. Composite/Cementum Interface

Composite/cementum interface roughness did not differ significantly among the composites at baseline (*p* > 0.05). There was a significant increase in Ra values after hygienic treatment with APPSB and APPCC in all composite groups (*p* < 0.05), whereas USS did not increase surface roughness significantly. No significant differences were registered among the composites after each type of hygienic treatment.

## 4. Discussion

In this study, we assessed the effects of ultrasonic scaling and air-powder polishing on the roughness of the enamel, three different nanocomposites, and composite/enamel and composite/cementum interfaces. The tested null hypotheses were as follows: there would be no differences in surface roughness (1) among the composite resins treated using different hygienic procedures, (2) among the three hygienic procedures for each composite resin and enamel, or (3) among the investigated surfaces, i.e., the composite surface, composite/enamel interface, and composite/cementum interface.

Based on the results obtained, the first hypothesis was partially accepted, as the mean roughness did not differ significantly between Premise and Herculite Ultra after hygienic procedures. However, it was rejected for Harmonize, since it exhibited the roughest surface among all composites after all hygienic procedures. The second hypothesis was rejected, since the hygienic method had a significant impact on the surface roughness of the enamel and composite materials. The third hypothesis was rejected as there was a significant difference in the roughness composite surface and the roughness of restoration margins.

Periodontal diseases are highly prevalent among the general population [2,3,40], and many patients receive hygiene maintenance therapy at regular intervals [11]. While this therapy effectively removes biofilm and staining, it may destroy superficial tooth structures and increase the roughness of hard tooth tissues [32]. Although many studies have aimed to define the critical level of restorations and hard tooth tissues roughness, this value remains undetermined. However, it is widely accepted that the threshold surface roughness for plaque retention is 0.2 μm [41,42,43]. Therefore, in our study, we considered this threshold to be clinically acceptable. Surface roughness was assessed with a contact stylus profilometer as this had been done in several studies [30,42,44,45].

In our study, we finished and polished composite restorations with aluminum oxide disks, as they have been shown to achieve a high-quality, smooth composite surface [19,31,46]. Composite roughness after finishing and polishing with aluminum oxide disks was 0.19 ± 0.12 μm, while in a study by Babina et al. this value was 0.09 ± 0.05 μm [31]. This difference is probably due to the longer working time in the latter study; finishing and polishing was performed until visible gloss was achieved (157 ± 15 s). We polished the specimens with medium, fine, and extra-fine grit discs for 10 s each [19], and all tested materials exhibited acceptable surface roughness at baseline (below or near the clinically acceptable 0.2 μm threshold). However, there was an increase in surface roughness after hygienic procedures in all groups. These results agree with those from other studies that have shown that all types of prophylactic treatment led to an increase in composite surface roughness [11,14,18,47,48].

The effects of hygiene maintenance procedures on surface roughness depend on the type of prophylaxis method [12,14,49] and the settings used [4,50], the type of composite material [18,49], and the number of treatments [47].

Ultrasonic scaling and air-powder polishing are widely used in periodontal prophylaxis [4,22].

Ultrasonic piezoelectric scalers have been shown to produce a significantly smoother surface than that produced by magnetostrictive device [17,51]. This is why we used a piezoelectric scaler in our study. The effect of ultrasonic instrumentation on surface roughness is material-dependent [14,16]. Despite their superior clinical performance compared with other composite materials [26], nanocomposites are also prone to surface damage resulting from ultrasonic treatment [15,18]. In the present study, ultrasonic scaling significantly affected the composite surfaces of Herculite Ultra and Harmonize, while the effect on the Premise surface was insignificant. These differences might be attributed to the nature of the evaluated materials (i.e., differences in the size and content of filler particles). The large size of pre-polymerized filler particles (30–50 μm) in Premise may result in both high initial roughness and slight alteration of the surface by ultrasonic scaling. Herculite Ultra with 1-μm-sized pre-polymerized filler particles exhibited the smoothest surface before and after ultrasonic scaling, although this treatment increased Ra values two-fold. According to Mourouzis, the presence of pre-polymerized organic fillers was related to greater alterations in surface roughness after sonic instrumentation [16]. However, Premise and Herculite Ultra both contain pre-polymerized filler particles and still exhibited different patterns of surface roughness alteration; therefore, we can assume that the size of the pre-polymerized particles is also an important factor.

Apart from the restorative material, the device settings may also influence the degree of surface damage. It has been shown that the following factors should be considered during hygienic treatment: lateral force, power setting, shape of the working tip, and angulation [4,52]. In our study, all specimens were treated similarly with a SATELEC piezoelectric scaler under water cooling, 10 cycles for 10 s each, at a low power setting (level 3), at 15–20° to the surface.

Another commonly used method of dental deposit removal is air-powder polishing [19,20]. It has been reported that air powder treatment increases the surface roughness of both crown and root surfaces [12,24] and restorative materials [11,53]. However, in a study by Salami et al., air-powder polishing did not affect the roughness of the enamel and radicular surfaces and did not produce rougher composite surfaces than the polished ones [21]. The potential harm of this procedure seems to depend on technical parameters such as consistency, abrasive size and shape, and the mode of application (distance, angulation, and treatment time) [24].

A broad range of powders differing in cleaning effectiveness and surface abrasion is available for use with air-powder polishers [50]. The greater the particle size and hardness are, the more abrasive the powder is [11]. To date, among the polishing powders used, sodium bicarbonate, glycine, and calcium carbonate are the most common [22,54]. In our study, we used calcium carbonate and sodium bicarbonate powders with the same particle size (65 μm) and found that both types of air-polishing powders significantly affected composite surfaces. There was no significant difference in the surface roughness after cleaning procedures with both sodium bicarbonate and calcium carbonate.

Apart from the powder type and characteristics of powder particles, there are other possible factors influencing surface quality after prophylaxis. These may include the amount of pressure [50], polishing time, angulation, and working distance [13,22,48]. In the present study, however, these factors remained constant, and their influence was minimized. Specimens were air-polished for 10 cycles of 10 s each at 4 bars of pressure at a 60-degree angle and 5.0 mm distance. Apart from the fact that this polishing mode was used in many other studies, it is also similar to that used in the clinical setting [50].

As well as ultrasonic treatment, air-powder polishing produces different extent of surface damage depending on the type of restorative material [19,49]. According to Pelka et al., nanocomposites with different composition may experience different surface changes after the prophylaxis procedure using various air powders [55].

In the present study, both types of air-polishing powders significantly affected the surfaces of all tested nanocomposites. No differences were found between the Ra values of Herculite Ultra and Premise, while Harmonize showed the roughest surface among the composites after both air-powder polishing treatments. The air-powder treatment can abrade the resin matrix, exposing composite filler particles [19,53]. Besides, it has been reported that powder particles can abrade the filler phase of composite resin materials [20]. As for the size of the abrasive, it should be smaller than the composite particle size to achieve a smoother surface [56]. Harmonize contains the smallest particles among the tested composites (nanometric spherical 30-nm conglomerates consisting of Zirconia (5 nm) and colloidal silica (20 nm)). We hypothesize that these particles may be easily removed from the composite surface resulting in greater roughness.

To the best of our knowledge, there have been no studies on the comparison of ultrasonic and air-powder hygiene maintenance procedures regarding composite surface roughness. We found no differences between these prophylaxis methods for Premise, while for the other composites (Herculite Ultra and Harmonize) the surface treated using air-powder techniques was significantly rougher compared to ultrasonic instrumentation.

Hygiene maintenance procedures may affect not only the composite surface itself, but also the margins of restorations by producing gap formation, surface porosity, microleakage [20,32,57], destruction of the adhesive layer [47,58] and even debonding of the restoration [20].

Composite/cementum and composite/enamel interfaces were shown to be rougher compared to the resin surface after finishing and polishing of the restoration [31]. In our study, we observed greater Ra values at the interfaces both before and after prophylaxis treatment. We hypothesize that the interface roughness after professional dental prophylaxis may result from the increase of roughness of each of the contacting surfaces (enamel, cementum, and composite resin) as well as from the damage to the adhesive layer.

There are controversial findings in the literature on the influence of prophylactic techniques on the enamel surface. According to Gerbo et al., SEM showed no statistically significant increase in enamel surface roughness after air polishing for the equivalent of a 15-year recall program [33]. Similar results were reported in a study by Salami et al., who found that sodium bicarbonate jet did not modify the superficial roughness of the enamel [21]. Ultrasonic instrumentation also showed slight influence on the enamel, as concluded by Andrei et al. [47]. However, a study by Yildrim et al. concluded that both ultrasonic and air-abrasive methods increased enamel roughness. Additionally, ultrasonic scaling caused greater damage to the treated surface compared to the air-abrasive technique with sodium bicarbonate powder [12]. Fratolin et al. showed the damaging potential of air-abrasive treatments for the enamel. This effect was observed for both calcium carbonate and sodium bicarbonate powders [59]. In the present study, hygienic treatment was a significant factor for enamel roughness; however, the increase in Ra values was mild and significant only in the bicarbonate group (1.13 μm and 1.4 μm at baseline and after hygienic treatment, respectively). Ultrasonic scaling and calcium carbonate powder did not increase surface roughness significantly. The differences between the studies may be explained by the different methods used to evaluate surface roughness, different prophylaxis procedure settings, and the comparison with the control specimens instead of the same specimens after treatment. Regarding composite/enamel interface, ultrasonic scaling significantly affected the roughness of Herculite Ultra/enamel and Harmonize/enamel interfaces. Ra values at the Premise/enamel interfaces were not influenced by ultrasonic instrumentation, probably due to minor damage to both adjacent surfaces (enamel and Premise material) caused by this procedure, as mentioned above. Air-polishing significantly increased composite/enamel Ra values for all composites irrespective of the powder used.

According to the literature, the cementum surface may also be damaged by hygiene maintenance procedures [12,24,34]. We did not assess the influence of professional hygiene techniques on the cementum surface itself, only on the composite/cementum interface. There were no significant differences in the surface roughness of composite/cementum interfaces in all composite groups after ultrasonic scaling, possibly due to comparable influence of this cleaning procedure on both surfaces (cementum and composite resin). Air-polishing significantly increased Ra values for all composites at composite/cementum interface irrespective of the powder used.

The limitations of this in vitro study include the use of only a contact stylus profilometer, which examines the surface along certain paths and does not allow assessment of the entire area; no comparison between different settings (angulation, distance, time); and no assessment of cementum surface roughness itself before and after instrumentation. Further research may focus on the evaluation of the amount of hard tooth tissues and restorative materials removed during prophylactic treatment as well as biofilm growth on the surfaces and restoration margins treated with different prophylaxis techniques.

Based on our findings, professional dental prophylaxis in the cervical area should be carried out with caution. Ultrasonic scaling and air-powder polishing in the area of Class V restorations should be avoided whenever possible. Subsequent repolishing of the roughened restorations and margins might be practical.

## 5. Conclusions

Within the limitations of this in vitro study, the following conclusions can be drawn: all tested prophylactic techniques increased the roughness of composite surface and composite/cementum and composite/enamel interfaces; the extent of composite surface damage is material-dependent; air-powder polishing with both calcium carbonate and sodium bicarbonate produced a greater increase in surface roughness of composite resin and restorations margins than ultrasonic scaling; hygiene maintenance procedures slightly affected enamel roughness.

## Figures and Tables

**Table 1:** Composition of the resin composite materials used in this study.

Resin Composite	Manufacturer	Filler Type	Filler Loading, % by Weight
Premise	Kerr, Scafati, Italy	Barium-aluminum-borosilicate glass (mean particle size 0.4 μm); fumed silica nanofiller (20 nm); prepolymerized filler (≈20–30 μm)	84
Herculite Ultra	Kerr, Scafati, Italy	Barium-aluminum-borosilicate glass (mean particle size 0.4 μm); fumed silica nanofiller (50 nm); prepolymerized filler (≈1 μm)	78
Harmonize	Kerr, Scafati, Italy	Barium-aluminum-borosilicate glass (mean particle size 0.4 μm); aggregated zirconia/silica cluster filler (2–3 μm) comprised of 20 nm spherical fumed silica and 5 nm zirconia particles	81.5

**Table 2:** Mean Ra values (μm) of the composite surface and composite/enamel and composite/cementum interfaces before and after hygiene maintenance procedures, M (σ).

Substrate		USS		APPCC		APPSB	
Surface	Composite	Baseline	Post treatment	Baseline	Post treatment	Baseline	Post treatment
CR	Premise	0.23 (0.12) ^a^	0.31 (0.06) ^abc^	0.24 (0.14) ^a^	0.40 (0.06) ^c^	0.24 (0.13) ^a^	0.41 (0.06) ^c^
	H. Ultra	0.13 (0.04) ^a^	0.26 (0.04) ^b^	0.15 (0.07) ^a^	0.40 (0.07) ^c^	0.14 (0.06) ^a^	0.43 (0.16) ^c^
	Harmonize	0.18 (0.15) ^a^	0.40 (0.11) ^c^	0.23 (0.19) ^a^	0.81 (0.08) ^d^	0.21 (0.03) ^a^	0.82 (0.06) ^d^
Total		0.18 (0.12)	0.32 (0.09) *	0.20 (0.14)	0.54 (0.21) *	0.19 (0.09)	0.55 (0.22) *
CR–E	Premise	1.2 (0.21) ^e^	1.22 (0.14) ^ef^	1.29 (0.15) ^e^	1.48 (0.22) ^f^	1.22 (0.10) ^e^	1.50 (0.22) ^f^
	H. Ultra	1.05 (0.29) ^e^	1.46 (0.40) ^f^	0.92 (0.22) ^e^	1.61 (0.38) ^fg^	0.94 (0.23) ^e^	1.69 (0.42) ^fg^
	Harmonize	0.92 (0.14) ^e^	1.32 (0.19) ^f^	0.96 (0.11) ^e^	1.96 (0.58) ^g^	0.98 (0.10) ^e^	2.00 (0.62) ^g^
Total		1.06 (0.24)	1.33 (0.28) *	1.06 (0.23)	1.68 (0.46) *	1.05 (0.20)	1.73 (0.48) *
CR–C	Premise	1.3 (0.19) ^e^	1.22 (0.27) ^e^	1.18 (0.22) ^e^	1.35 (0.08) ^f^	1.11 (0.13) ^e^	1.38 (0.06) ^f^
	H. Ultra	1.18 (0.16) ^e^	1.26 (0.31) ^ef^	1.04 (0.16) ^e^	1.40 (0.21) ^f^	1.01 (0.12) ^e^	1.42 (0.21) ^f^
	Harmonize	1.11 (0.24) ^e^	1.27 (0.28) ^ef^	1.10 (0.23) ^e^	1.29 (0.15) ^f^	1.07 (0.18) ^e^	1.29 (0.08) ^f^
Total		1.20 (0.21)	1.25 (0.29)	1.11 (0.21)	1.35 (0.16) *	1.06 (0.15)	1.36 (0.14) *

^a–g^—different letters indicate statistically significant differences among groups; *—significant differences between pre- and post-treatment values (without division into particular composites); CR—composite resin surface; CR–E—composite/enamel interface; CR–C—composite/cementum interface; USS—ultrasonic scaling; APPCC—air-powder polishing using calcium carbonate; APPSB—air-powder polishing using sodium bicarbonate.

**Table 3:** Mean Ra values (μm) of the enamel before and after hygiene maintenance procedures, M (σ).

Substrate	USS		*p* Value	APPCC		*p* Value	APPSB		*p* Value
	Baseline	Post treatment		Baseline	Post treatment		Baseline	Post treatment	
Enamel	1.01 (0.22)	1.18 (0.18)	0.058	1.11 (0.28)	1.32 (0.25)	0.083	1.13 (0.29)	1.40 (0.31)	0.047 *

*—significant differences between pre- and post-treatment values; USS—ultrasonic scaling; APPCC—air-powder polishing using calcium carbonate; APPSB—air-powder polishing using sodium bicarbonate.

## Data Availability

The data are available from the corresponding author upon reasonable request.
